# Effect of folic acid and vitamin B12 on the expression of PPARγ, caspase-3 and caspase-8 mRNA in the abdominal aortas of rats with hyperlipidemia

**DOI:** 10.3892/etm.2013.1076

**Published:** 2013-04-24

**Authors:** FENG-HUA LV, JIAN-ZHI GAO, QING-LEI TENG, JIN-YING ZHANG

**Affiliations:** 1 Department of Cardiology, The First Affiliated Hospital of Xinxiang Medical University, Xinxiang, Henan 453100;; 2 School of Basic Medical Sciences, Xinxiang Medical University, Xinxiang, Henan 453003;; 3 Department of Cardiology, The Third Affiliated Hospital of Xinxiang Medical University, Xinxiang, Henan 453100;; 4 Department of Cardiology, The First Affiliated Hospital of Zhengzhou University, Zhengzhou, Henan 450000, P.R. China

**Keywords:** hyperlipidemia, PPARγ, caspase-8, caspase-3, folic acid

## Abstract

Hyperlipidemia may lead to endothelial injury, due to its effects on homocysteine and vascular endothelial growth factor in the serum, and the mRNA expression levels of peroxisome proliferator-activated receptor-γ (PPARγ), and caspase-3 and -8 in the vascular wall. In order to prevent and mitigate the high-fat state that results from endothelial injury, this study examined the effect of folic acid (FA) and vitamin B_12_ (VB12) on the expression of PPARγ and caspase-3 and -8 mRNA in the abdominal aortas of rats with hyperlipidemia. Sixty 4-week-old healthy male Sprague Dawley rats were randomly divided into five groups (each n=12): the normal control (NC), high-fat diet (HL), FA, VB_12_ and FA+VB12 groups. Following one week of adaptive feeding, the FA, VB12 and FA+VB12 groups were subject to the intraperitoneal injection of FA (0.5 mg/day), VB_12_ (0.05 mg/day) and FA+VB_12_ (0.5 mg/day and 0.05 mg/day), respectively, while fed a high-fat diet. The rats in the NC group were injected intraperitoneally with 0.9% NaCl solution (0.5 ml/day) and fed a normal diet, whereas those in the HL group were fed a high-fat diet only. A reverse transcription-polymerase chain reaction (RT-PCR) assay demonstrated that at the end of week 12, the FA treatment had effectively increased the PPARγ mRNA level, while reducing the caspase-3 and -8 mRNA levels, compared with the high-fat diet treatment (P<0.05). The effect of FA on the expression of PPARγ and caspase-3 and -8 was enhanced when used in combination with VB_12_ (P<0.05). These results revealed that the application of FA, alone or in combination with VB_12_, improves and mitigates the high-fat state that results from endothelial injury.

## Introduction

With the continuous improvement in the standard of living worldwide, the population of individuals with hyperlipidemia has expanded. Hyperlipidemia damages multiple organ systems, particularly the cardiovascular system, and eventually leads to atherosclerosis (AS), a complex vascular inflammatory disease. The common pathological process of AS is the formation of an atheromatous plaque, accompanied by the disorder of lipid metabolism and damage to endothelial function (1). Hyperglycemia, hypertriglyceridemia, hypercholesterolemia and hyperinsulinemia have been correlated with a higher risk of heart and cerebrovascular diseases (2). There are >15 million fatalities worldwide due to cardio- and cerebrovascular diseases every year, making these diseases the leading cause of mortality (3). There is therefore a fundamental requirement to decrease the levels of glucose and lipids.

In recent years, peroxisome proliferator-activated receptors (PPARs) have been observed to be expressed in regions of atherosclerotic injury (4). Following activation, PPARs are able to protect endothelial cells and improve the function of the endothelium through a variety of pathways. PPARγ agonists have been demonstrated to inhibit the expression of CD68, monocyte chemoattractant protein-1 (MCP-l), vascular cell adhesion molecule-1 (VCAM-l) and tumor necrosis factor-α (TNF-α) in rats with low-density lipoprotein (LDL) receptor-deficiency, in addition to hindering the monocyte-macrophage accumulation in the vascular wall (5). Furthermore, PPARγ downregulates the expression of the MCP-l receptor, C-C chemokine receptor type-2 (CCR2) (6) and thereby reduces the inflammatory reaction in local vessels. PPARγ ligands may be mediated by nitric oxide (NO), which effectively inhibits the expression of matrix metalloproteinase (MMP)-9 in macrophages (7). As a member of the nuclear receptor superfamily, PPARγ participates in the formation of fats, and lipid and glucose metabolism. In addition, it has a major impact on vascular biology and inflammation, particularly in the development of atherosclerosis. PPARγ is able to protect blood vessels by preserving vascular endothelial function, regulating inflammatory cytokine and adhesion molecule expression, inhibiting macrophage activation, promoting the reversal of cholesterol transport, inhibiting vascular smooth muscle cell proliferation, and migrating and stabilizing atherosclerotic plaques (8).

Caspases are a group of cysteine proteases with aspartic acid-specific restriction sites, which cause apoptosis through protein lysis (9). The caspase family of proteases may directly initiate the disintegration of apoptotic cells, and is thus important in the molecular mechanisms of apoptosis (10). The caspases serve as convergent points in a number of apoptotic pathways, and the majority of caspase family proteases act as significant promoters or effectors of apoptosis (11). At present, 14 types of caspases are recognized, and these are divided into three categories: i) apoptosis-initiating factors, including caspase-8; ii) apoptosis effectors, including caspase-3, which may be activated by an upstream promoter; iii) those mainly involved in cytokine-mediated inflammatory response and play a supporting role in the death receptor-mediated apoptosis pathway. Once activated, the caspases act on specific substrates and induce biochemical and morphological changes in the cells, resulting in apoptosis. Caspase-3 is the primary effector molecule and has critical functions (12). Animal experiments have demonstrated that the apoptosis of endothelial cells may be induced by homocysteine (Hcy), and that a large number of apoptotic endothelial cells are present in plaques. Thus, Hcy-induced endothelial cell apoptosis may be closely correlated with caspases. In this process, caspase-8 acts as the apoptosis-initiating factor, while caspase-3 acts as the apoptotic effector. However, the molecular mechanism(s) that initiates and triggers the caspase-induced apoptosis of endothelial cells has not yet been elucidated, and futher investigations are therefore required.

The aim of the current study was to examine the effect of folic acid (FA) and vitamin B_12_ (VB12) on the mRNA expression of PPARγ, and caspase-3 and -8 in the abdominal aortas of rats with hyperlipidemia. The hyperlipidemic rat model was established with the artificial feeding of a high-fat diet. The changes in the level of PPARγ mRNA expression, and the effects of caspase-3 and -8 on endothelial cell apoptosis were examined via a reverse transcription-polymerase chain reaction (RT-PCR) assay. The protective effects of FA and VB_12_ on the vascular endothelial cells was studied through drug intervention, in order to provide new methods and a theoretical basis for the clinical treatment of cardio- and cerebrovascular diseases.

## Materials and methods

### Experimental animals

Sixty healthy 4-week-old male Sprague Dawley (SD) rats, weighing 110±10 g, were provided by the Experimental Animal Center of Henan Province (Zhengzhou, China). The rats were randomly divided into five groups (each n=12): the normal control (NC), high-fat diet (HL), FA, VB_12_ and FA+VB_12_ groups. Following one week of adaptive feeding, the rats in the FA, VB_12_ and FA+VB12 groups were fed with a high-fat diet, and injected intraperitoneally with FA (0.5 mg/day), VB12 (0.05 mg/day) and FA+VB_12_ (0.5 mg/day and 0.05 mg/day), respectively. The rats in the NC group were injected with 0.9% NaCl solution (0.5 ml/day) and fed a normal diet, whereas those in the HL group were fed a high-fat diet only. During the experiment, one rat treated with FA, two rats treated with VB_12_ and two rats treated with FA+VB12 died. The remaining rats survived and were considered to be in a good condition. The study was approved by the ethics committee of Xinxiang Medical University (Xinxiang, China).

### Serum preparation and blood lipid determination

At the end of week 12, the rats were weighed and injected intraperitoneally with 10% chloral hydrate (0.3 ml/100g). Following anesthesia, blood samples were taken from the hearts of the rats, and were centrifuged at 5000 × g for 5 min at 4°C. The supernatant was stored at −20°C, prior to blood lipid determination. The levels of total cholesterol (TC), triglycerides (TG) and LDL and high-density lipoprotein (HDL) cholesterol were quantified using an Architect CI8200 Automatic Biochemistry Analyzer (Abbott Laboratories, Abbott Park, IL, USA).

### RT-PCR assay of PPARγ and caspase-3 and -8 mRNA levels in the abdominal aorta

Total RNA was extracted from the blood vessel using TRIzol^®^ reagent (Invitrogen Life Technologies, Carlsbad, CA, USA). The extracted RNA was transformed to cDNA by RT-PCR, using three pairs of primers: PPAR-γ, forward: 5′-CACAAGAGCTGACCCAATGGT TGCTG-3′ and reverse: 5′-CGCAGATCAGCAGACTCT GGGTTC-3′ (product size: 345 bp); caspase-8, forward: 5′-AATGTTGGAGGAAAGCAATC-3′ and reverse: 5′-CAT AGTCGTTGATTATCTTCAGC-3′ (product size: 380 bp); and caspase-3, forward: 5′-TGTCATCTCGCTCTGGTACG-3′ and reverse: 5′-AAATGACCCCTTCATCACCA-3′ (product size: 279 bp). The PCR mixture contained 20 *μ*l reaction solution, including 2.0 *μ*l cDNA, 10 *μ*l 1X Taq reaction buffer and 0.2 *μ*M primer (0.4 *μ*l forward and reverse primers, respectively; Bioer Technology Co., Ltd., Hangzhou, China). The PCR amplification was performed at 94°C for 46 sec, 58°C for 46 sec and 72°C for 45 sec (35 cycles). The PCR products were separated using 1.5% agarose gel electrophoresis.

### Statistical analysis

The mRNA levels were quantitatively analyzed using the high-resolution Motic Images Advanced Software (Motic, Xiamen, China). The data are presented as the arithmetic mean value ± standard deviation. The Student’s t-test and analysis of variance (ANOVA) were conducted using SPSS 13.0 software (SPSS, Inc., Chicago, IL, USA) for the comparison of two groups and multiple groups, respectively. P<0.05 was considered to indicate a statistically significant difference.

## Results

### Blood lipid levels in adult rats

The TC, TG and LDL and HDL cholesterol levels were 0.92±0.85, 3.93±0.23, 0.73±0.12 and 0.49±0.01 mmol/l, respectively, in the rats of the HL group. Of these results, the TC, TG and LDL cholesterol levels were significantly higher in the HL than in the NC group (P<0.05), whereas the HDL level was significantly lower in the HL than in the NC group (P<0.05). There were no statistically significant differences observed between the blood lipid levels of the rats in the HL group and the rats in the groups that received drug intervention, i.e. FA, VB_12_ and FA+VB_12_ (Table I). This indicated that the application of FA and/or VB_12_ had no significant effect on the blood lipid levels in adult rats.

### PPARγ and caspase-3 and -8 mRNA expression in the vascular walls of adult rats PPARγ mRNA expression

**Figure 1.**
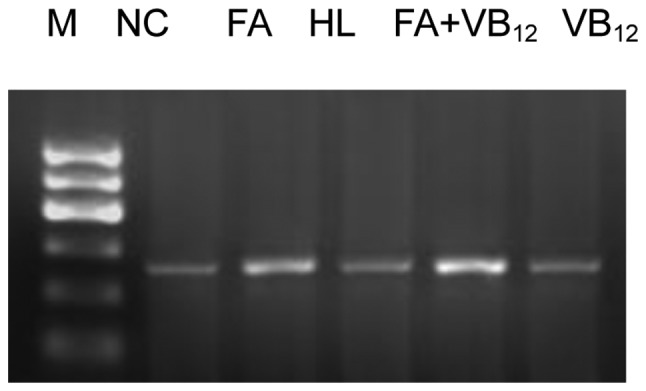
PPARγ mRNA expression levels in the abdominal aortas of adult rats following 12 weeks of treatment with folic acid (FA) and/or vitamin B_12_ (VB_12_), combined with a high-fat diet. M, DNA marker; NC, normal control group; HL, high-fat diet group.

The levels of PPARγ mRNA expression in the rats of the FA and FA+VB_12_ groups were significantly higher than those of the HL and VB_12_ groups (P<0.05). In addition, a statistically significant difference was observed between the PPARγ mRNA expression levels in the rats of the FA and FA+VB_12_ groups (P<0.05). The level of PPARγ mRNA expression in the rats was significantly different between the NC and HL groups (P<0.05), but not between the VB_12_ and HL groups (P>0.05; Fig. 1).

### Caspase-3 and -8 mRNA expression

**Figure 2.**
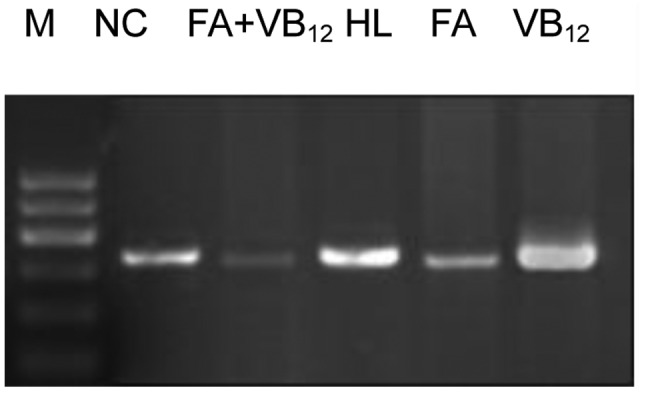
Caspase-8 mRNA expression levels in the abdominal aortas of adult rats following 12 weeks of treatment with folic acid (FA) and/or vitamin B_12_ (VB_12_), combined with a high-fat diet. M, DNA marker; NC, normal control group; HL, high-fat diet group.

**Figure 3.**
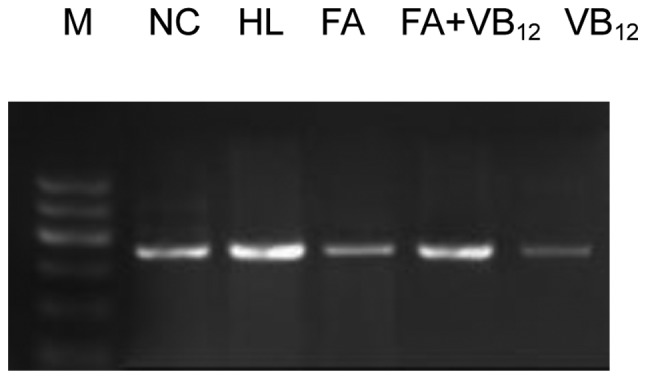
Caspase-3 mRNA expression levels in the abdominal aortas of adult rats following 12 weeks of treatment with folic acid (FA) and/or vitamin B_12_ (VB_12_), combined with a high-fat diet. M, DNA marker; NC, normal control group; HL, high-fat diet group.

**Figure 4.**
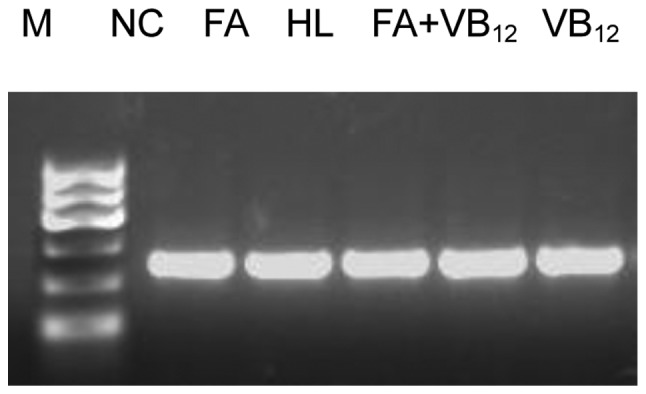
β-actin mRNA expression levels in the abdominal aortas of adult rats following 12 weeks of treatment with folic acid (FA) and/or vitamin B_12_ (VB_12_), combined with a high-fat diet. M, DNA marker; NC, normal control group; HL, high-fat diet group.

The caspase-3 and -8 mRNA expression levels in the rats of the FA and FA+VB_12_ groups were significantly lower than those in the rats of the HL and VB_12_ groups (P<0.05). Furthermore, a significant difference was observed in the levels of expression of these caspases between the FA and FA+VB_12_ group rats (P<0.05). No significant differences were observed in the levels of caspase-3 and -8 mRNA expression between the rats of the VB_12_ and HL groups (P>0.05), although the levels were significantly higher in the HL group than in the NC group (P<0.05; Figs. 2–4).

## Discussion

There are a number of risk factors correlated with coronary heart disease (CHD), including immutable factors, including age, gender and a family history of premature CHD, and major modifiable factors, including hypertension, hyperglycemia, hyperlipidemia, smoking and obesity. In the present study, a animal model of CHD was established using SD rats fed a high-fat diet, and FA and VB_12_ interventions were administered to the rats, in order to explore the causative factors of CHD and the relevant disease mechanisms, in addition to the potential clinical application of FA and VB_12_(13). The results demonstrated that the rats with hyperlipidemia (group HL) had significantly higher serum TC, TG and LDL cholesterol levels, and lower serum HDL cholesterol levels compared with the control rats (group NC, Table I). These results indicated that the rats with hyperlipidemia were at risk of obesity, leading to endothelial cell damage and eventually the induction and acceleration of CHD occurrence and development.

By contrast, the FA, VB_12_ and FA+VB12 treatments did not exert any significant effects on the TC, TG and LDL and HDL cholesterol levels in the rats with hyperlipidemia (Table I), which demonstrated that FA and VB_12_ are not suitable for the clinical treatment of obesity or blood lipid metabolism.

With regard to the effect of FA and VB_12_ on the levels of caspase-3 and -8 mRNA expression in the abdominal aorta of adult rats, it was revealed that the caspase-3 and -8 mRNA levels increased in the HL and VB_12_-treated rats, whereas the levels were reduced in the rats treated with FA and FA+VB12, in comparison with the control group. These observations suggested that FA is able to effectively inhibit Hcy-induced endothelial cell apoptosis, thereby protecting the integrity of endothelial function and preventing endothelial injury. Studies have indicated that there are at least three apoptotic signaling pathways that are related to the activation of caspases: i) the mitochondrial/cytochrome *c* pathway; ii) the death receptor pathway and iii) the endoplasmic reticulum pathway (14,15). The signal for apoptosis may be conveyed through one or more channels, thereby activating caspases and eventually inducing apoptosis. It was revealed by Schuerwegh that NO is important in the TNF-α-induced apoptosis of bovine chondrocytes, and it has been confirmed that there is a close interrelation between TNF-α and caspase-3, as well as between caspase-3 and NO, in the process of apoptosis (16). High Hcy-induced endothelial cell apoptosis is commonly accompanied by changes in vascular endothelial growth factor (VEGF), endothelin (ET) and NO, which affect the expression of apoptosis-related caspase enzymes, and further induce the apoptosis of endothelial cells. FA is able to reduce the Hcy concentration in the blood and change the VEGF, ET and NO levels. As a result, this affects the expression of caspase enzymes, and ultimately has an impact on the apoptosis of endothelial cells (17).

In the present study, the levels of PPARγ mRNA were observed be significantly lower in the HL and VB_12_-treated rats than in the control group, which was indicative of endothelial cell damage and dysfunction (Table II). By contrast, the PPARγ mRNA levels increased significantly in the rats treated with FA and FA+VB_12_ compared with those in the HL group, which suggested that FA effectively reduced the Hcy level and prevented endothelial cell dysfunction, thus protecting the integrity of endothelial function and preventing endothelial injury. However, further investigations are required, in order to study the detailed molecular mechanisms of these processes, and to determine whether FA and VB_12_ are PPARγ agonists. PPARγ agonists are capable of activating PPARγ, thus protecting the endothelium, and affecting the complex changes in the cytokines and inflammatory factors that occur following endothelial damage, in the process of AS (5,6).

In conclusion, the removal of CHD-promoting factors and the protection of endothelial cell function are required for the treatment of CHD. This study demonstrated that FA intervention and treatment was effective in reducing the Hcy concentration in the serum of rats fed with a high-fat diet, and with elevated VEGF levels and AS. In addition to an intensive lipid-lowering treatment, the supplementary application of an appropriate quantity of FA and VB_12_ may effectively protect the function of the vascular endothelium, thus contributing to the prevention and treatment of CHD.

## Figures and Tables

**Table I.** Blood lipid levels in the serum of adult rats following 12 weeks of treatment with FA and/or VB_12_, combined with a high-fat diet.

Group	n	TC (mmol/l)	TG (mmol/l)	LDL cholesterol (mmol/l)	HDL cholesterol (mmol/l)
NC	12	0.40±0.05	1.65±0.98	0.50±0.05	0.87±0.10
HL	12	0.92±0.85^a^	3.93±0.23^a^	0.73±0.12^a^	0.49±0.01^a^
FA	11	0.92±0.10	3.83±0.31	0.76±0.15	0.48±0.04
FA+VB_12_	10	0.91±0.06	3.89±0.23	0.69±0.10	0.52±0.08
VB_12_	10	0.90±0.08	3.91±0.21	0.74±0.07	0.48±0.04
F statistic	-	102.898	292.381	12.833	66.800
P-value		<0.05	<0.05	<0.05	<0.05

a P<0.05 compared with the normal control (NC) group. There was no significant difference in the blood lipid levels between each group with drug intervention and the high-fat diet (HL) group (P>0.05). FA, folic acid; VB_12_, vitamin B_12_; TC, total cholesterol; TG, triglycerides; LDL, low density lipoprotein; HDL, high denisty lipoprotein.

**Table II.** Relative mRNA expression levels (gray value ratio) in the abdominal aortas of adult rats following 12 weeks of treatment with FA and/or VB_12_, combined with a high-fat diet.

Group	n	Caspase-8/β-actin	Caspase-3/β-actin	PPARγ/β-actin
NC	12	0.45±0.07	0.43±0.07	0.66±0.04
HL	12	0.70±0.12^a^	0.70±0.13^a^	0.44±0.04^a^
FA	11	0.40±0.07^b^	0.40±0.07^b^	0.71±0.02^b^
FA+VB_12_	10	0.25±0.02^b^^c^	0.25±0.02^b^^c^	0.80±0.01^b^^c^
VB_12_	10	0.71±0.02^d^	0.72±0.12^d^	0.46±0.04^d^
F statistic		51.962	52.031	254.531
P-value		<0.05	<0.05	<0.05

a P<0.05 compared with the normal control (NC) group;

b P<0.05 compared with the high-fat diet (HL) group;

c P<0.05 compared with the folic acid (FA) group;

d P>0.05 compared with the HL group. VB_12_, vitamin B_12_. PPARγ, peroxisome proliferator-activated receptor γ.
